# Gender-based violence and associated factors among private college female students in Dessie City, Ethiopia: mixed method study

**DOI:** 10.1186/s12905-022-02076-3

**Published:** 2022-12-12

**Authors:** Selamawit Gebrie, Yitbarek Wasihun, Zinet Abegaz, Natnael Kebede

**Affiliations:** 1 Department of Reproductive and Family Health, School of Public Health College of Medicine Health Sciences, Wollo University, Dessie, Ethiopia; 2 Department of Health Promotion, School of Public Health College of Medicine Health Sciences, Wollo University, Dessie, Ethiopia

**Keywords:** Gender-based violence, Factors, Students, Ethiopia

## Abstract

**Introduction:**

Violence against girls and young women, mostly those in educational settings, has been gaining increasing attention. School-based gender-based violence represents a serious obstacle. As a result, it would be a threat to the achievement of the sustainable development goals, strive for gender equality in all our programs, right from the planning stages, to make sure we are as equitable as possible. Little was studied to explore reasons, opinions, and perceptions towards gender-based violence. Also, studies that are conducted on private college female students are limited. Important evidence about underlining reasons for gender-based violence against private college students will be explored. Therefore, to assess the prevalence of gender-based violence and associated factors among Private college female students in Dessie City, Ethiopia, 2021.

**Methods:**

A facility-based mixed method concurrent triangulation study design was conducted among 435 randomly selected Private college female students in Dessie City. Self-administered questionnaire and an in-depth interview were used to collect the data. The collected data were cleaned and entered into Epi data and analyzed using a statistical package for social science. Descriptive statistics were conducted and the results were reported using frequency, and percentile. Binary logistic regression was performed to identify associated factors. Adjusted odds ratios with 95% confidence intervals and *p* values < 0.05 were used to explain statistically significant associations. Qualitative data were transcribed, translated, and analyzed manually using thematic analysis.

**Results:**

The study showed the prevalence of gender-based violence was 251 (62.6%) (CI 0.512–0.683) Private college female students in Dessie city Administration. age less than 20 years and 20–24 years, [AOR = 0.19, 95% CI (0.03–0.92)] and [AOR = 0.106, 95% CI (0.02–0.44)], tight family control, [AOR = 6.14, 95% CI (1.38–7.1)], family discussion on RH and related personal issue [AOR = 0.091, 95% CI (0.03–0.27)], Witnessed father abuse mother at childhood; [AOR = 4.04, 95% CI (1.36–12.1)], had drunkenness female or boyfriend; [AOR = 5.12, 95% CI (1.58–16.5)] had significant association with gender-based violence.

**Conclusions:**

In this study, the high prevalence of gender-based violence among Private college female students is higher as compared to others. This is because the life of young girls is being abandoned as a result of gender-based violence, such as dropout from their education, unwanted pregnancy, abortion, sexually transmitted infections, and psychological disturbance, which decreases the productivity of girls. This is totally against the strategy and consensus of universal education for women and girls and adolescent health stated in the sustainable development goal.

## Introduction

Gender-based violence as it's described by the united nation general assembly is any form of violence that causes physical, psychological, and sexual impairment for the victim this includes acts like coercion, and illogical denial of liberty, which can be occurred in public or private life [1]. Gender-based violence may take many forms: Physical abuse, for example, hitting, beating/stinging/pushing, destroying her assets, threatening or hurting her by using different weapons, and denying access to health care [2]. Psychological/emotional abuse includes scaring her, taking her children away from her, harassing her, controlling her behavior and where she spends her money, isolating her from family/friends, and threatening to harm those important to her people. Verbal abuse, such as verbal abuse, use of other words to lower self-esteem, and humiliation in front of others [3, 4]. Sexual abuse, such as forcing them to engage in unwanted sexual activity, becoming pregnant or having an abortion, and exposing them to sexually transmitted diseases. Spiritual abuse includes demeaning her spiritual beliefs and preventing her to worship her choice. Financial abuse is like preventing access to a family resource [2, 5].

Violence against girls and women is a major violation that results in gender inequality. GBV is present in every country in the world. Globally one in three women experience some form of violence in childhood, adolescence, or adulthood period. It is becoming a major public health problem because of its acute and chronic effects on women's health. Acute effects include morbidity and mortality as a result of physical abuse [6]. Long-term effects of violence include chronic pain, gynecological morbidity, sexually transmitted diseases (including HIV), obesity, hypertension, smoking, depression, and suicide [7].

Violence against girls and young women, mostly those in educational settings, has been gaining increasing attention. Declines in academic performance and dropping out of school at higher frequencies are major problems for victims of gender-based violence than those not violated. In African countries prevalence of GBV among college-age women is very high. Ethiopia is primarily a traditional society with rigid gender norms and cultural values [8].

GBV challenges adolescents' capacity of dealing positively with their sexuality and their ability to minimize unintended pregnancies and sexually transmitted infections, including HIV.GBV also causes Psychological and social consequences to victims [9, 10].

Violence is the result of the complex interaction of individuals, relationships, and social, cultural, and environmental factors. Individual-level factors include personal characteristics and behaviors that increase the chance of being a victim or perpetrator. The relationship factors include relations with peers, intimate partners, and family members [11]. In community contexts in which social relationships are embedded—such as schools, workplaces, and neighborhoods, societal factors which increase the rate of violence include cultural norms that support violence as an acceptable way to resolve conflicts, norms that support male dominancy, economic and social environment and women empowerment [9]. Girls often didn't report gender-based violence because of a lack of reporting systems between students' schools and health facilities. In addition, there are poor systems in schools to support them after disclosure [10].

Enormous studies were conducted globally as well as in our country on gender-based violence and associated factors but as far as my search most of the studies focus on the magnitude and associated factors with GBV. Studies which explore reasons, opinions, perceptions, and beliefs are too small, and also studies that are conducted on Private college female students are scarce particularly in Dessie city.

Previous studies were conducted using a cross-sectional study design with limited qualitative methods and were unable to see the experience of female college students with GBV conducting with a mixed method is more important to get multiple determinants and comprehensive results for intervention. Therefore, this study will provide important evidence about perceptions, opinions, and reasons that increase the magnitude and associated risk factors of GBV on private college students by its qualitative component. And will contribute a lot to minimizing the problem of shortage of evidence related to the issue understudy area. The finding of the study will give insights for School administrators to build a strong collaboration with community leaders, policymakers, law enforcement bodies, women advocates, and other stakeholders.

## Materials and methods

### Study design and period

A mixed-method concurrent triangulation study design was conducted from November to April 2021.

### Population

All Private college female students in Dessie City Administration were taken as the source population. Private College female students in Dessie City administration during the data collection period were considered as the study population.

### Sampling method and sample size determination

The sample size was determined by using a single population proportion formula by taking the following assumptions: Prevalence of lifetime Gender-based violence (physical or/and sexual violence) was 59.9% with a 95% confidence interval, 5% marginal error, 10% for non-response rate in the study conducted in Hawassa town female college students [7], a confidence interval (CI) of 95%, marginal error of 5%.

n = (Z α/2)2 P (1 − P)/W2, n = (1.96)2*0.599 (1 − 0.599)/(0.05)2, n = 3.8416 * 0.599 * 0.401/0.0025 = 369. Finally, by adding 10% non-responses, the total sample size was 406.

The sample size for the qualitative method was determined by the degree of saturation.

Simple random sampling using a computer random number generator was used to select study participants in each college using the student roster from the register to select 406 students. For the Qualitative part, the sample size was taken when the level of saturation was reached. We say the sample is Saturated when adding more participants to the study was not result in additional perspectives or information. So our sample size was 12 undertaken for in-depth-interview.

### Operational definitions

**Violence:** is defined as, "The intentional use of physical force or power, threatened or actual, against oneself, another person, or against a group or community, that either result in or has a high likelihood of resulting in injury, death, psychological harm, or deprivation." [12].

**Gender-based violence** "Gender-based violence" For this study is defined as physical and/or sexual that targets girls based on their gender [13].

**Lifetime GBV:** defined as ever-faced physical and/or sexual that targets girls based on their gender in their lifetime of college [13].

**Physical violence:** is any form of a violent act that can result in physical harm including mild form (slapping, and punching) or severe form (kicking/drugging, beating/hitting with any object, burning/chocking, and threatening using a knife or a gun, etc) against women or girls [14].

**Sexual violence:** is defined as acts that are done to a girl by the intentional use of physical force or power, intimidation, or threatening (making to fear) to have sex or to engage in acts of sex without the consent of the girl. It includes Completed Rape, Attempted Rape, and Sexual Harassment [13].

**Completed rape**: is defined as any non-consensual penetration of the vagina, penetration obtained by physical body harm, by threatening or deception, or when the victim is incapable of giving consent [15].

**Attempted rape:** is defined as a trial to have sexual intercourse without consent by physical body harm, by threatening or deception, or when the victim is incapable of giving consent but without actual penetration of the vagina [15].

**Sexual harassments**: these are unwanted sexual behaviors including physical contact, verbal comments, jocks, questions, and suggestions that are intentionally done on women or girls [16].

**Family control**: different types of measurement actions taken by parents against children's behavior [16].

**Tight family control** parents always know close friends, what you doing outside the home, and your plan for the coming day [16].

**Average family control** parents sometimes know close friends, what you doing outside the home, and your plan for the coming day [16].

**Poor/loose family control** parents never know close friends, what you doing outside the home, or your plan for the coming day [16].

**Family income: **the amount of money incurred per month at the family level in Ethiopia [17]

- Good- ≥ 8900 ETB
- Average- ≥ 2800–8900 ETB
- Poor- < 2800 ETB.

### Data collection procedure and quality assurance

The questionnaire was prepared in English and translated into the Amharic version. Finally, it was back-translated into English by another person to ensure consistency. A pre-test was done at Rada College Kombolcha Town. The data collection tool included 8 family histories, 8 sexual experiences, 8 physical violence statuses, 11 sexual violence statuses, 12 substance uses, 6 school conditions, 6 perceptions towards GBV, and 11socio-demographic questions. Qualitative data was collected by using in-depth interviews and an observational checklist.

### Rigors and trustworthiness of the study

The researcher and advisors discussed on objectives of the study, confidentiality of information, contents of the open-ended semi-structured questionnaire, and data quality management before data collection started. Few participants were enrolled once the data has been transcribed to review the ideas for transcription verification, for them to think the investigator is going to present a true picture from their perspective.

To ensure authenticity, all data were included in the final report to allow the readers to see the basis upon which conclusions were made. Rigor was attained through strict attention to detail, adhering to procedures, and consistency and accuracy throughout the research process. A peer review was conducted to ensure credibility. The data, analysis, interpretations, and conclusions were continuously peer-reviewed who are taking training on qualitative research. One session with peer researchers from the same backgrounds was used to check the consistency between the analysis of data and thematic development.

An audit trail was created throughout the data collection, transcriptions, a clear description of the analysis process, and interpretation of data. The audio-taped interviews were not destroyed until the member check and transcription verification. Moreover, Reflexivity involved self-awareness, and bracketing was used to set aside any preconceived ideas regarding lived experience to ensure confirm the ability of results that can reduce the researcher's bias.

After data collection, the principal investigators transcribed the audio-recorded data in the participant's local language into written form and then back-translated to English, then the data was coded, categorized, and analyzed for ease of interpretation. Therefore using a tape recorder, careful probing, verbatim transcription, interviewing up to reaching saturation point, peer review, audit trail, and considering disparity were used to keep the trustworthiness of the data of the study.

### Data processing and analysis

Data were checked for completeness and consistency after that it was coded and entered into EPI-data v 4.6.0.2, and then it will be exported to SPSS 23 statistical software for process and analysis. Different frequency tables, graphs, and descriptive summaries were used to describe the study variables. Binary logistic regression analysis was used to see the significance of the association between dependent and independent variables. Bi-variable and multivariate were computed to see the association between GBV and selected independent variables. In bivariable logistic regression analysis variable with a *p* value of 0.20 or less was entered for multivariate logistic regression analysis to adjust for potential confounders, then p value less than 0.05 was considered a predictor for GBV, and an adjusted odds ratio with 95% CI was considered to see the strength and significance of the association. The adequacy of the final model was checked using the Hosmer and Lemeshow Goodness of fit test with a *p* value was 0.9. Multi colinearity test was checked using variance inflation factors (VIF) with 4.12.

Qualitative data were analyzed using thematic analysis. Before analysis, all the collected data were transcribed into English. The interview was transcribed, and performed by the researcher. Data transcribed by the principal investigator was read several times to evaluate critically to get the concept and group into themes based on the concept they contain. So responses were categorized under each theme and sub-theme. An investigator analyzed data to answer the study objective and write a report based on categorized themes. Quotes were used to highlight each category and show association with each theme.

## Results

### Socio-demographic characteristics of participants

A total of 401 female students participated in this study. The mean age of respondents was 22.83 ± 3.64 years. The majority of stud participants 296 (73.8%) were enrolled in the degree program and 165 (41.1%) were in the second year. About 132 (32.9%) participants live with family and 101 (25%) with female friends. About three fourth of participants score more than 3.0 cumulative previous average grade point (Table 1).

**Table 1:** Socio-demographic characteristics of private college female students in Dessie City Administration, Ethiopia, 2021

Variables	Category	Frequency	Percent (%)
Age	< 20 years	108	26.9
	20–24 years	199	49.6
	> 24 years	94	23.4
Religion	Orthodox	164	40.9
	Catholic	3	7
	Protestant	61	15.2
	Muslim	173	43.1
Ethnicity	Tigray	34	8.5
	Amara	210	52
	Afar	83	20.7
	Oromia	43	10.7
	Others	31	9.5
Childhood residence	Urban	200	49.1
	Rural	201	50.1
With whom currently living	Alone	57	14.2
	With my family	132	32.9
	With husband/boyfriend	34	8.5
	With female friends	101	25.2
	With relatives	75	18.7
	Others	2	0.5
Are you currently married or have a boyfriend	Yes, married	41	10
	Yes, boyfriend	129	32.2
	No	231	57.6
Partner educational status (n = 170)	No formal education	2	1.2
	Complete grades 1–8	5	2.9
	Complete grades 9–12	25	14.7
	Grade 12 and above	138	81.2
Employment status of partner (n = 170)	Student	56	32.9
	Employed	51	30
	Un employed	5	2.9
	Self-employed	58	34.1
Educational program	Diploma	31	7.7
	Degree	295	73.5
	Master	75	18.7
Education level	First-year	73	18.2
	Second year	165	41.1
	Third year	133	33.2
	Fourth and above	30	7.5
Last semester result	2.00–2.75	58	14.5
	2.76–3.00	48	12
	> 3.00	295	73.6
Where do you place your current grade status	Good and above	122	30.9
	Average	257	64.1
	Poor	22	5.5

### Prevalence of gender-based violence (GBV)

GBV was computed by aggregating the findings of 'any form of sexual violence and 'any form of physical violence acts in their lifetime. The magnitude of GBV (sexual or/and physical violence) among the study participants was found to be very high. It was reported by 251 (62.6%) of the respondents in their lifetime. And also physical and sexual violence were reported at 62.3% and 39.2% respectively.

### Family history

Among the study participants, 216 (53.6%) of them have parents living together, and the educational status of parents, 124 (30.9%) of their fathers had completed grade 12 and above and 190 (47.4%) of their mothers had no formal education. The majority of the students 282 (70.3%) perceived that they were receiving enough money according to their demand for their education and other expenses and 233 (58.1%) reported that their families were close to them and have support if needed. Similarly, 242 (60.3%) of the students perceived that they have tight family control 0.226 (56.3%) participants witnessed parental violence as a child (i.e. their mothers were beaten by husbands or male partners) (Table 2).

**Table 2:** Family history of private female college female students in Dessie City Administration, 2021 Ethiopia

Variables	Category	Frequency	Percent (%)
The living condition of the mother and father	Live together	216	53.9
	Divorced/separated	68	17.0
	Only mother alive	43	10.7
	Only father alive	28	7
	Both of them are not alive	46	11.3
Father educational status	No formal education	98	24.4
	Grades 1–8 complete	94	23.2
	Grades 9–12 complete	85	21.2
	Above grade 12	124	30.9
Mother educational status	No formal education	190	47.4
	Grades 1–8 complete	102	25.4
	Grades 9–12 complete	32	8
	Above grade 12	77	19.2
Family members support	Yes	233	58.1
	No	168	41.9
Enough money according to the demand	Yes	282	70.3
	No	119	29.7
Perceived family income	Good	43	10.7
	Average	256	63.8
	Poor	102	25.4
Perceived Family control	Tight	242	60.3
	Average	118	29.4
	Loose/free	41	10.2
Witnessed parental violence as a child	Yes	226	56.4
	No	175	43.6

### Substance use and related behaviors

Chewing chat was found to be the most substance abused 131 (32.7%), followed by alcohol 103 (25.7%). Whereas 12 (3%) of the study participants smoked cigarettes/tobacco in their lifetime and almost all participants does not experience drug use like cocaine. One hundred thirteen (28.2%) of the respondents reported that they have either male or female friends who are drunk currently.

### Sexual experiences

Among the total study participant 182 (45.3%) respond that they had experienced sexual intercourse. Of those 82 (45%) of them were before the age of 18 and 100 (54.2%) of them were greater than 18 at the time of first sexual intercourse. Of those participants who have sexual experience, 119 (65.4%) of them didn't know the age of their sexual partner.

One hundred seventeen (64.3%) of the sexually active respondents reported that they have experienced more than one sexual partner in their lifetime. And 168 (92.3%) of these sexually active students have only one sexual partner currently. And more than half of them (53.3%) were not willing in their first sexual intercourse. Peer pressure (37.4%), false promises (28.6%), financial support (16.5), and being drunken (15.4%) were the most listed reasons for not being willing the first sexual intercourse with participants (Table 3).

**Table 3:** Sexual experience of private college female students in Dessie City administration, 2021, Ethiopia

Variables	Category	Freq	Percent (%)
Ever had sexual intercourse	Yes	182	45.3
	No	219	54.6
Age at first sexual intercourse	Less than 15 years	16	8.7
	15–18 years	66	37
	Greater than 18 years	100	54.3
Age of first sexual partner (n = 182)	< 18	8	4.4
	18–24 years	9	4.9
	> 24	46	25.3
	I don’t know	119	65.4
Willingness at first sexual intercourse (n = 182)	Yes	85	46.7
	No	97	53.3
Reason for not willing in first sexual intercourse (N = 97)*	Family pressure/marital	1	1.1
	Peer pressure	34	37.4
	Threatened	12	13.2
	False promise	25	28.6
	For financial support (money)	15	16.5
	To pass exam	12	13.2
	Made me drunken	14	15.4
Having more than one sexual partner currently (n = 182)	Yes	14	7.7
	No	168	92.3
Number of sexual partners experienced (n = 182)	One	65	35.7
	Two	83	45.6
	Three	21	11.5
	Four and above	13	7.1
Discussion of RH issues with family	Yes	177	44.1
	No	224	55.9

N.B * multiple responses can't be added up to 100%

### Physical violence

Six items were used to measure physical violence. These are purposeful acts (**YES** responses) of pushing and slapping for mild form; and beating, kicking/dragging, burning/choking, and threatening for the severe form of physical violence as reported by the respondents.

Physical violence was computed by aggregating these six items using SPSS 23 transformation functions. At least one **YES** response among the six items qualifies the respondent for being faced with any form of physical violence.

Out of the total study participants, 250 (62.3%) respondents experienced at least one form of physical violence. 209 (52.1%) of the participant experienced mild physical violence (i.e. Slapped, Kicked/dragged, Beaten with a fist, Pushed), and 39 (9.7%) of the respondents experienced severe forms of physical violence (i.e. Choked or burnt and Threatened with a gun, knife, or another weapon) (Table 4).

**Table 4:** Prevalence of physical violence in Private college female students in Dessie city administration, 2021, Ethiopia

Variables	Category	Frequency	Percent (%)
Slapped or threw something at you	Yes	177	44.1
	No	224	55.9
Pushed or shoved	Yes	191	47.6
	No	210	52.4
Beaten with a fist or other something	Yes	155	38.7
	No	246	61.3
Kicked, dragged, or beaten u	Yes	99	24.7
	No	302	75.3
Choked or burnt on purpose	Yes	33	8.2
	No	368	91.8
Threatened with a gun, knife, or another weapon	Yes	137	65.8
	No	264	34.2

### Perpetrators of physical violence

Relatives and students were the most listed perpetrators of physical violence (68.8%) and (69.1%) respectively (Table 5).

**Table 5:** Perpetrator of physical violence among private female college female students in Dessie city Administration 2021, Ethiopia

Variables	Frequency	Percent (%)
Boyfriend/husband	65	26
Family member	89	35.6
Another relative	172	68.8
Teacher	89	35.6
Student	173	69.1
Stranger	41	16.4
Other	25	10

N/B multiple responses can't be added up to 100%

### Consequences of physical violence

**Fig. 1:**
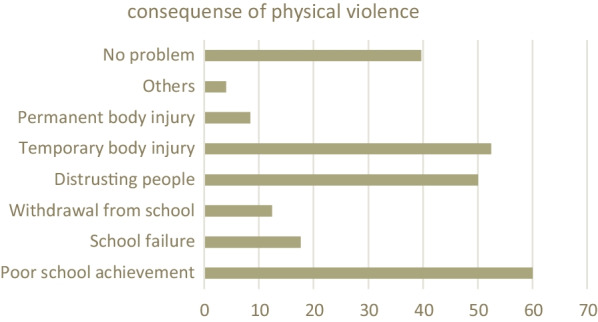
Reported consequences of physical violence in college female students of Dessie city Administration, 2021 Ethiopia

Among the victims of physical violence, 150 (60%) were reporting poor school achievement, more than half of respondents (52.4%) report temporary body injury and 125 (50%) reported that they had a tendency of disgusting people following the physical violence they have experienced. While 39.6% of physically violated participants report no problem due to violence (Fig. 1).

### Sexual violence

Among the study participant, the prevalence of any form of sexual violence was 157 (39.2%); One hundred thirty-nine (34.7%) college students responded that they had faced unwelcome touch or sexual harassment. Attempted rape 72 (18%) and complete rape 55 (13.5%) were reported.

### Frequency and tendency of reporting about sexual violence

Out of these 55 complete rape cases, more than half (50.9%) of the respondent experience it at once in their lifetime. Only 13% of these rape cases informed the condition of their family and only 5.45% reported to the police. The majority of respondents list shame/guilt 40 (72.2%), as a reason for not reporting violence.

### Consequences of sexual violence

Most listed health-related consequences of completed rape were injury around the genitalia 35 (63.6%) and swelling around the genitalia 26 (42.2%), fear and anxiety were the most reported psychological consequences of rape 43 (81.1%), poor achievement or failure from school 36 (67.9%) and rejection from friends 28 (52.8%) were listed as social consequences following rape.

### School related factors

Among study participants, 19.95% of them believed that gender-based violence was common on their campus. The majority of the study participants do not see experiences that reflect equity and equality between men and women in college (72.8%), and 76.6% of them also do not feel safe at their school toward GBV. Almost all participants (93%) revealed that they had no access to programs to learn about and discuss GBV and related issues (Table 6).

**Table 6:** School-related factors of college female students in Dessie city Administration 2021, Ethiopia

Variables	Category	Frequency	Percent (%)
Ever face GBV in this collage	Yes	80	19.95
	No	321	80.04
Collage society knows about violence against female	Yes	29	7.2
	No	165	41.1
	Have no idea	207	51.6
Are there rules to punish the perpetrator of GBV	Yes	20	5
	No	136	33.9
	Have no idea	245	61.1
Is there an Experience that reflects equity and equality between men and women	Yes	109	27.2
	No	292	72.8
Feeling safely related to GBV at school	Yes	94	23.4
	No	307	76.6
Programs to learn and discuss GBV	Yes	28	7
	No	373	93.1

### Perception towards GBV

Majority of participants in the study, (87.5%) perceive that sexual violence is a major problem for female students in colleges. from those participants (91%) perceived that it is preventable problem. almost all participants reported that sexual violence is a criminal violation (84%), creating awareness among females, males, and families and empowering females were listed as means of prevention mechanism by most of the participants. most of the participants get sexual-related information from video, film, the internet (67.4%), mass media (51.4%), and friends (43%) (Table 7).

**Table 7:** Perception of Private college female students towards GBV in Dessie city administration 2021

Variables	Category	Frequency	Percent (%)
Perceive GBV against the female as a problem	Yes	351	87.5
	No	50	12.5
Perceiving this problem as punishable	Yes	337	84
	No	64	16
Perceiving those problems as preventable	Yes	365	91
	No	36	9
Perceived mechanisms to prevent those problems*	Legal enacting	183	45.9
	Create awareness of female	266	66.5
	Create awareness of male	257	64.4
	Create awareness of family	213	53.4
	Empowering women	232	58.1
	Strengthen clubs	91	22.8
	Stop girls appeal dressing	163	40.9
	Other	10	2.5
Perceived reasons for GBV*	Girls dressing style	313	78.4
	Male dominancy	251	62.9
	Low negotiation power of girls	234	58.6
	Drinking alcohol	206	51.6
	Smoking cigarette	62	15.5
	Chewing chat	76	19
	Loss family control	127	31.8
	Other	9	2.3
Source of information about sexuality*	No information	71	17.8
	The school (teacher)	66	16.6
	Health worker (health center)	110	27.6
	Family	49	12.3
	Friend	172	43.1
	Mass media	205	51.4
	Video,film,internet	269	67.4
	Other	22	5.5

N.B * multiple responses can't be added up to 100%

### Factors associated with gender-based violence

In bivariable analysis, nine variables namely, age, educational program, Witnessed father abusing mother, Family living together, family support, family control, Family Discuss RH issues, Age of sex partner, and friend who drunk were candidate variables for multivariable logistic regression at a *p* value of less than 0.20.

In multivariable logistic regression, six of the nine variables were significantly associated with GBV at a 5% level of significance. The significant predictors of GBV were: tight family control (*p* < 0.05), discussion RH with family (*p* < 0.001), Witnessing a father abuse mother (*p* < 0.05), having a friend who drank alcohol (*p* < 0.01), educational program (*p* < 0.05) and age of student (*p* < 0.05) had a statistically significant association with GBV.

Female college students whose age less than 20 years and 20–24 years, the odds of GBV were reduced by 81% and 89.4% respectively when compared > 24 years [AOR = 0.19, 95% CI (0.03–0.92)] and [AOR = 0.106, 95% CI (0.02–0.44)].Female college students who had tight family control were 6.14 more likely to have GBV when compared with Loss family control [AOR = 6.14, 95% CI (1.38–7.1)].female college students who have a family discussion on RH and related personal issues, the odds of GBV were reduced by 91.8% when compared with who do not discuss RH and related issues with family [AOR = 0.091, 95% CI (0.03–0.27)].female college student who had Witnessed father abuse mother at childhood were 4.04 times more likely to had GBV when compared with who hadn't Witnessed abuse; [AOR = 4.04, 95% CI (1.36–12.1)].a female College students who had a drunken female or boyfriend were 5.12 times more likely to GBV when compared with who hadn't a drunken friend; [AOR = 5.12, 95% CI (1.58–16.5)] (Table 8).

**Table 8:** Factors associated with gender-based violence among female college students Dessie, Ethiopia 2021

Variable	Category	GBV		COR (95% CI)	AOR (95% CI)	*p* value
		Yes	No			
Family support	Yes	136	97	1.58 (1.02–2.34)	0.86 (0.307–2.45)	0.79
	No	115	53	1	1	
Family living together	Yes	130	86	0.42 (0.19–0.89)	0.59 (0.12–2.86)	0.50
	Divorced	38	30	0.35 (0.15–0.82)	0.45 (0.78–2.106)	0.28
	Only mother	27	16	0.46 (0.18–1.19)	0.52 (0.59–4.55)	0.55
	Only father	20	8	0.69 (0.23–2.04)	0.44 (0.18–11.0)	0.62
	Both not live	36	10	1	1	
Age of sex partner	< 18	4	4	0.32 (0.07–1.13)	0.11 (0.013–1.04)	0.52
	18–24 years	5	4	0.40 (0.10–0.60)	0.78 (0.91–6.82)	0.83
	> 24 years	30	16	0.60 (0.28–1.26)	0.55 (0.18–1.67)	0.29
	Not known	90	29	1	1	
Education program	Diploma	17	13	0.24 (0.09–0.64)	0.84 (0.72–9.80)	0.89
	Degree	171	125	0.26 (0.13–0.50)	0.24 (0.071–0.84)	0.02*
	Master	63	12	1	1	
Age of respondent	< 20	65	43	0.16 (0.07–0.35)	0.19 (0.03–0.92)	0.039*
	20–24	101	98	0.10 (0.05–0.22)	0.106 (0.02–0.44)	0.002*
	> 24	85	9	1	1	
Family control	Tight	175	69	4.22 (2.21–8.07)	6.14 (1.38–7.1)	0.017*
	Medium	58	51	1.89 (0.96–3.79)	1.372 (0.30–6.23)	0.68
	Loss	18	30	1	1	
Freely Discuss RH With the Family	Yes	63	102	6.34 (4.05–9.90)	0.091 (0.03–0.27)	0.000*
	No	188	48	1	1	
Witnessed father abuse mother	Yes	175	47	0.19 (0.12–0.30)	4.04 (1.36–12.1)	0.012*
	No	76	103	1	1	
Had a friend who drinks alcohol	Yes	95	18	4.46 (2.26–7.76)	5.12 (1.58–16.5)	0.006*
	No	156	132	1	1	

NB: *significant at *p* < 0.05

## Discussion

Gender-based violence among college female students in Dessie city administration was found to be a very common problem. The prevalence of gender-based violence (physical or/and sexual) was found to be 62.6% (CI 0.512–0.683) in a lifetime. The prevalence of any form of sexual violence was reported to be 39.2% (CI 0.325–0.434) in a lifetime whereas that of any form of physical violence was 62.3% (CI 0.597–0.723) in a lifetime.

A study conducted on Awasa College female students in 2006 revealed that the prevalence of gender-based violence in a lifetime is 59.9% which is consistent with these findings [18]. Whereas the magnitude of overall gender-based violence in our study is higher than that of Debre Marcos university, North West Ethiopia which is 36% [19]. This might be due to most college students living in a rental house with friends or alone. This may increase the risk of violence. Another possible explanation for the differences might due to sociocultural nature variations of the study populations and the study settings, such as access to substances. On the other hand, our finding on sexual violence is consistent with the 2012 study conducted in Bahir-Dar town among female college students revealed to be 37.3% in a lifetime [20].

In our study, completed rape and attempted rape in a lifetime were reported by 13.5% (CI 0.126–0.154). and 18%(CI 0.176–0.232)respectively which are comparable results with a study on sexual coercion conducted among female students of Wollo University in 2018 revealed to be 13.7% and 21.4% respectively [21]. But our findings were higher than the findings from female high school students in Debark which is 8.8% for completed rape and 12.4% for attempted rape [22]. These differences might be due to the study settings (community or institution and rural or urban). Another possible explanation might be due to punishment and follow up the difference between this area for that rapper person.

Our findings on completed rape were lower than the community-based studies conducted around Gondar in Northern Ethiopia which is 19.2% [23]. This might be due to the contexts of forced rape (domestic violence) and it could be because our study is institution based and the prevalence is among survivors so exposed to underestimation.

In this study, physical violence showed that lifetime prevalence was reported by 62.3% (CI 0.597–0.723). which is comparable with the finding in Debre Markos town menkorer high school (66.1%) [24] and higher than the study conducted on female high school students in wolita sodo and the study conducted among college female students of Mekelle town, the prevalence of lifetime physical violence was 56.3% and 46.3% respectively [25, 26]. This difference might be due to age differences and the sociocultural difference between the target population.

Our study has assessed some associated factors which are defined as contributing factors to gender-based violence. Gender-based violence was significantly associated with age, those age groups less than 20 and 20–24 were 81% and 90% times less likely to experience gender-based violence as compared to the age group greater than 24 but in another study conducted at Mekele town age group greater than 24 were reported as greater risk of experiencing gender-based violence [26]. In study reveals that having boyfriend or female friends who drink is associated with gender-based violence; this finding is comparable with a study conducted in Menkorer high school in Debre Markos town northwest Ethiopia [24].

"Chewing chat is a common phenomenon in the area where I grow up I was chewing with my mother at home because my parents have a chat farm. When I come to this college it is very difficult for me to stop it and I can't afford to buy chat so I was obligated to go with people who need a girl to make a coffee and chew chat with them. Then I start drinking different alcohols with them one day I was drinking too much and was raped by the one who I chew chat with" a 22 years 3rd-year student.

"Drinking Tela is like drinking water in our community children are not prevented to drink tela which is why I'm familiar with such a drink. After joining this college I'm not drinking Tela but on special occasions like friends' birthdays, weekends and some holidays I and my friends drink beer, wine, and the like to make fun". A 21-year second-year student.

On the other hand, those who freely discussed RH issues with family members were 91.8% times less likely to suffer from GBV. This is comparable to the study findings in Bahir Dar private colleges and Debre Markos University [19, 20]. This might be due to a lack of adequate information and experience in how to handle RH-related situations. In our study witnessing domestic violence as children had a 4.04-fold increased risk of them experiencing gender-based violence. This is consistent with a study conducted on female college students at Awasa [18].

My father and mother were always in conflict because he always comes drunk at the night. if she refuses his act he kicks and insults her, I asked him to stop but he slaps me and always I frustrate him in the nighttime." 1^st^ year BSc student.

Our study reveals that those having tight family control were 6.14 times more at risk of experiencing gender-based violence as compared with those who have lost family control; which is consistent with the study conducted at Debre Markos university [19]. This might be due to the need for young girls to test new things which increases the risk of GBV.

"When they live with their family students are under close supervision but when they try to manage themselves especially those from the rural area they are exposed to different bad behaviors which exposes them to gender-based violence.

Those female students who were under tight family control needed to test new things when they get some relaxation they try everything including engaging in sexual relationships which is very abusive. I know one girl in our college that fired from school as a result of her absence because she was spending her time with bad guys. 29-year administrative staff."

In this study different problems were reported as a result of physical violence, this includes distrusting people (50%), temporary body injury (52%) and poor school achievement (60%), other problems like school failure, withdrawal from school, and permanent bodily injury were also widely reported. Relatives, teachers, and students were the most listed perpetrator of physical violence.

"I was one of the best students in the lower class when I join high school it was too far from home and I was suffering from guys on the road, they destruct me and I always think about how can I escape from them and I lost my concentration on a class that is the reason why I didn't score point that allows me to join government universities". 3rd year student.

"My friend has no one eye as she told me one day her father and mother were not in the house, her brother and she quarreled each other and he pushed her and she failed on the stone". 2nd-year student.

"Those physical violence's are considered as punishing the child for his misbehavior if it is performed by a family member, relatives, and occasionally by neighbors. I think nobody considers the bad outcomes.me and my sister and brothers encountered slaps, kicked by stick many times". 19 years old First-year student.

"Boys who are sitting on the road are the one who holds your hand and sometimes may give you a fist if you did not respond in the way they like". 3rd-year student.

In our study, the main reasons for not being willing in the first sexual experience among female students were peer pressure, false promises, financial support, and being drunk.

"When I was in grade 10 there was a boy who asked me to be his girlfriend repeatedly but I refused to be his girlfriend at the end of the year there was a party that is prepared at our school me and friends participated in that party, I was pressured by my friends to drink wine for the first time, I was drunk and dance with that guy, after all, I got myself with him". 20-year second-year student.

I know a girl who was forced to have sex repeatedly because of not have enough money for school fees and house rent, a 23-year 4th-year student.

Different health-related, social, and psychological problems following rape were reported among the victims, injury around the genitalia (63.6%), swelling around genitalia (42.2%), unusual vaginal discharge and abortion (34.5%), fear and anxiety (81%), self-blame (60%), poor school achievement (52.9%), rejection from friends (52.8%), withdrawal from school (43.4%)Other problems such as unintended pregnancy, STI including HIV/AIDS also widely listed

Abortion is common among college students even though most students who are not raped also have abortions many times. There is a nurse who always contacts me when our students get into abortion. As she told me their reason was that they don't have their income to have a child” (**35-year Academic dean).**

"Things are going in the wrong direction after I get raped, I don't know what to do, where to go I only cry day and night (crying) umm there was pain, fear shame dark feature, I was afraid when people see my eye I thought they know what is happening on me I was absent from school many times by giving different reasons to family, I can't explain what I feel at that time. it was horrible, it took me many times to move out from that feeling, I lost my school performance, my personality, and my dream …. I don't want to remember that time (crying again). such inhuman action must be stopped!! This may be the story of many girls who are not living their dreams too. It needs attention" 3rd-year student.

"I was raped by my brother's friend, I remember the day he was like a family in our house he always comes to our house to have lunch with us, and like one day my aunt was sick and my mother and brother took her to the hospital I was at home as always he comes to our home but nobody is there, he raped me, for about a month it was very pain full I can't even urinate" **20-year 2nd-year student.**

“I need to kill myself when I get raped by my father-in-law he used to rape me repeatedly; I can't tell my mother because she has no income to take care of my little brothers and me. I didn't trust any man; he kills my future, my hope, and my confidence. I was very depressed and not able to concentrate on my school'**s 21-year 3rd-year students.**

In our study sexual violence was perceived as a major and punishable problem by 87.5% & 84% of respondents. And also 91% of respondents believe it can be preventable. Creating awareness on girls (66.5%), male (64.4%),creating awareness on family (53.4%), empowering girls (58.1%) and stop girls appealing dressing (40.9%) were listed as a mechanism for prevention of sexual violence.

Presence of high information gap among the respondents as reported by 17.8% of them not getting any information concerning gender-based violence or other sexuality issues currently. On the other hand, the internet was reported as a source of information by most participants which exposes them to unnecessary information such as seeing pornographic films that aggravate violence.

“A 21 years girl stated that almost all girls including me do not consider unwelcome touches and jokes as violence. But they have an impact on our confidence and academic performance too. Because they limit girls from expressing their thought and opinion in front of other colleagues and being frustrated to ask questions. In general, it's very common for girls to be violated as a result of their gender."

"A29-year-old girl expresses that prevention of GBV requires strong commitment, and regulatory bodies should enforce private colleges to include gender issues in their strategic goals. And also to strengthen clubs, guidance, and counseling services to help females discuss sexuality and related issues and allow them to solve problems. In addition to this creating awareness and empowering girls is the best mechanism to prevent GBV.

"the 19-year-old girl explains that in my opinion, the cause for the increasing gender-based violence is male dominancy. Many other factors have a contribution but if we see the behavior of males starting from their home they are mostly dictators they abuse their sister, then when they go to the community they try to exercise that behavior”

"In my opinion, GBV is prevented by working on families because children exercise what they see in their home for example if the father respect and love his wife and children and vice versa they can learn how to respect each other this goes to the environment then to school and becoming their life principle” 32-year administrative staff.

In our study, we recognize that private colleges are not as favorable for females regarding gender-based violence. 20% of study participants face GBV in college, and more than half of participants explained that they don't have any idea whether the college society knows about violence that happens to females or not and the presence of rules and regulations to punish perpetrators of GBV. This is because the college is not engaged in different activities to secure females from violence.

"the 20-year girl states that most violence in the school is performed by teachers and male students. Especially teacher calls the name of a female student and joke in the class to embarrass her, or they may give incorrect exam results and ask her to go to the office if she refuses a sexual relationship. "

23-year girl "in my experience of 2 years in this college in every activity, males are dominantly participants I don't think equity and equality principles are applied for example in demonstration rooms male students, as well as lab assistants, don't give chance for girls to practice. Class schedules and different events in the school are programmed by males this is because girls are made not to speak loudly and are unable to make decisions.

“No one gives attention to such gender-related issues in private colleges because it needs additional resources like human, financial, even time and place there is not only money there is a shortage of rooms to facilitate such issues. In addition to that private colleges focus on areas that can increase their income.” Administration staff

In this study GBV is a significant problem among female students in private colleges, this is due to a lack of commitment in private colleges to incorporate gender issues into their system. Most participants have no idea about the presence of rules and regulations in the college to punish perpetrators which leads them not to share their problems with school society. Females were shown to be victims of various forms of sexual and physical violence which range from unwelcome comments and unwanted touch to unhuman physical punishment and forced sexual intercourse.

### Strength and limitation

A combination of quantitative and qualitative methods involvement of administrative staff in the qualitative method (IDI) might consider the strength of this study. Recall bias that might consider a limitation of this study.

## Conclusion

Gender-based violence among female private college students in the study area was found to be very high. However, it is among survivors and prone to underreporting. More than half of the respondents reported having faced some form of Sexual or/and physical violence in their lifetime.

Lifetime Gender-based violence was associated with female students who witnessed parental violence as a child, had drunk male or female friends, was aged less than 24, those who do not discuss RH issues with family and come from tight family control.

In the in-depth interview sessions, participants stressed cultural practices that favored males' dominance and aggression as a sign of courage and manliness. This is started at a home (family) level and distributed to the community and the failure of government officials in forcing private learning institutions to incorporate gender issues and unable to put legal actions into practice doubles the problem of GBV in higher learning institutions. There is a belief that the sole reason for a girl student to be violated is due to her appealing dressing style and her dependency on males due to different reasons such as to get academic and financial support. This is also supported by some female students' in-depth interviews. Such a high prevalence of gender-based violence in higher learning institutions (colleges and universities) is very shocking and intolerable. This is because the life of young girls is being abandoned as a result of gender-based violence, such as dropout from their education, unwanted pregnancy, abortion, STIs, and psychological disturbance, which decreases the productivity of girls. This is totally against the strategy and consensus of universal education for women and girls and adolescent health stated in the Sustainable Development Goal (MDG). Colleges should secure effective education or awareness strategies, organize and strengthen clubs, and youth-friendly services and work in collaboration with associations such as the Women and Youth Association. Expanding and strengthening information, education, and behavior change communication (IE/BCC) activities aimed at preventing gender-based violence at the college level as well as in the community.

## Data Availability

All the necessary data are included in the manuscript. An English version data collection tool and detailed operational definitions of the outcome variable are accessible at a reasonable request from the corresponding author**.**

## Abbreviations

AOR: Adjusted odds ratio
CI: Confidence interval
HIV: Human immune deficiency virus
IDI: In-depth interview
MOE: Ministry of education
OR: Odds ratio
STI: Sexual transmitted infection
SDG: Sustainable development goal
UNICEF: United Nations International Children's Emergency Fund
USA: United States of America
VIF: Variance inflation factor
WHO: World Health Organization
